# Reactive oxygen species activated by mitochondria-specific camptothecin prodrug for enhanced chemotherapy

**DOI:** 10.17305/bjbms.2022.7194

**Published:** 2022-04-17

**Authors:** Zhaopei Guo, Zian Wang, Ruifeng Liang, Huayu Tian, Xuesi Chen, Meiwan Chen

**Affiliations:** 1 State Key Laboratory of Quality Research in Chinese Medicine, Institute of Chinese Medical Sciences, University of Macau, Macau, China; 2 Key Laboratory of Polymer Ecomaterials, Changchun Institute of Applied Chemistry, Chinese Academy of Sciences, Changchun, China

**Keywords:** Camptothecin prodrug, reactive oxygen species, antitumor therapy

## Abstract

Camptothecin (CPT) has attracted much attention due to its potent antitumor activities. However, the undesirable physicochemical properties, including poor water solubility, unstable lactone ring, and severe adverse effects, limit its further application. In this study, two water-soluble prodrugs, CPT-lysine and CPT-arginine, were designed and synthesized by conjugating lysine or arginine with CPT, improving its solubility, pharmacokinetic properties, and tumor penetration. Importantly, the introduction of arginine into CPTR contributed to the mitochondria-specific delivery, which increased mitochondrial reactive oxygen species generation, induced mitochondria dysfunction, and enhanced cell apoptosis and in vivo anti-cancer effect. This strategy is believed to hold great potential for organelle-specific synergistic anti-tumor therapy.

## INTRODUCTION

Camptothecin (CPT), first isolated from the Chinese tree *Camptotheca acuminata*, was widely used in preclinical or clinical anti-cancer therapy, including lung, colon, and breast cancer, as the topoisomerase I inhibitor [1,2]. However, poor water solubility and the instability of lactone ring in the physiological environment limit the therapy efficacy of CPT [3-5]. Another bottleneck in the application of CPT is the high-dose requirement and systemic toxicity due to its non-specificity. Tumor-specific delivery, including delivery to tumor tissues, tumor microenvironment, cells, and subcellular organelles (mitochondria, lysosome, endoplasmic reticulum, etc.), has gained much attention and exhibits great potential for therapeutic efficacy improve [6].

Among them, mitochondria, as the major cellular powerhouse, plays an essential role in cancer cell survival, as well as cell invasion and metastasis. Therefore, this organelle has attracted wide attention in cancer therapy [7,8]. Mitochondrion showed different functions in cancer cells compared to normal cells, including different metabolic activity, reactive oxygen species (ROS) generation, and transmembrane potential [9-12]. Mitochondria are the main source of superoxide radicals, which provide the possibility of exceeding cytotoxic thresholds in cancer cells [13,14]. Therefore, the development of novel mitochondrial-targeted CPT derivatives and CPT-based drug delivery systems would contribute to the powerful cancer therapy based on improved drug stability, efficient drug delivery, mitochondrial disruption, and associated homeostasis imbalance.

At present, various mitochondria-targeted nanosystems are developed for improved cancer therapy [15-17]. The molecules containing positive charges were regarded as an efficient tactic in traversing mitochondria double membrane to achieve mitochondria-targeted delivery. For example, triphenylphosphonium bromide was used as a classical mitochondria-targeting agent [18]. Nevertheless, the extremely low loading efficiency and instability of CPT hindered its further applications [19]. Amino acids (lysine, glycine, arginine, etc.) as promoieties have drawn much attention as well, since they provide structural diversity and expanse of physicochemical properties for optimization in drug delivery [20,21].

For instance, Vale et al. designed and synthesized new bromothiazole derivatives with amino acids, and confirmed the antiproliferative activities against colon cancer cells [22]. Deshmukh’s team confirmed that the introduction of amino acids (aminobutyric acid and norvaline) to CPT was able to stabilize the lactone form and offer the platform for further drug delivery [23]. Etienne et al. synthesized a series of CPT-amino acids carbamate linkers with a convenient and facile approach, in which the intramolecular cyclization contributed to increased stability and drug potency [21]. Azzolini et al. used carbamate ester as a linker to introduce isoleucine, to increase absorption, reduce metabolism, and acquire higher drug concentrations of pterostilbene [24]. Among these amino acids, the positively charged amino acid arginine holds great potential in the design of both cell-penetrating and mitochondrial-targeted drug delivery systems [25]. On the other hand, the amphiphilicity of water-soluble amino acid-modified CPT prodrugs is beneficial for the construction of carrier-free drug delivery nanosystems (nanospheres, nanotubes, nanofibers, or nanowires) with high CPT loading content based on self-assembly of drugs [26]. For example, π-π stacking between the heterodimeric prodrugs 7-ethyl-10-hydroxycamptothecin (SN38) and taxane largely increased the stability of heterodimeric prodrugs and lipid carrier-based micelles, as well as the drug loading content [27].

**SCHEME 1:**
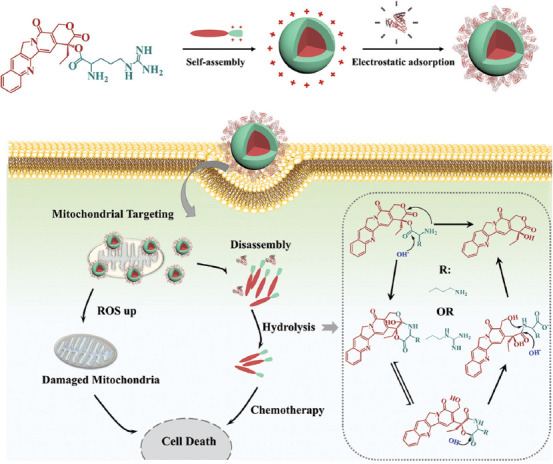
Schematic illustration of the anticancer mechanism of CPT prodrugs. CPT: camptothecin.

As shown in Scheme 1, positively charged amino acid-modified CPT prodrugs were synthesized (CPTK and CPTR) and assembled into nanoparticles to achieve improved cancer therapy based on increased drug stability, mitochondrial-targeted delivery, and deeper tumor penetration, with the following advantages exhibited. First, the improved water solubility and stability of CPTK and CPTR provided significant advantages over CPT for *in vivo* applications. Second, CPTR elevated ROS levels based on efficient drug penetration in mitochondria and induced mitochondria dysfunction. In addition, CPTR exhibited powerful penetration ability, contributing to *in vivo* cancer therapy. Thus, the novel CPT derivatives were supposed to have great potential in clinical anti-cancer therapy.

## MATERIALS AND METHODS

### Materials

Boc-Arg (Pbf)-OH and Boc-Lys (Boc)-OH were purchased from GL Biochem Ltd. (Shanghai, China). CPT was purchased from Dalian Meilun Biotechnology Co., Ltd. (Dalian, China). Trifluoroacetic acid (TFA) was obtained from Xiya Reagent Co., Ltd. (Linyi, China). EDC hydrochloride was provided by J&K Scientific Ltd. (Beijing, China). N-Hydroxysuccinimide and DMAP were supplied by Aladdin Co. Ltd. (Shanghai, China). 4’-6-diamidino-2-phenylindole was purchased from Sigma-Aldrich (St. Louis, MO, USA). Methyl thiazolyl tetrazolium (MTT) was obtained from Amresco (Solon, Ohio, USA). Annexin V-FITC apoptosis detection kit, ROS Assay Kit (DCFH-DA), Mitochondrial Membrane Potential Assay Kit with Rhodamine 123, Fluo-4 AM, and MitoTracker Red were obtained from Beyotime (Shanghai, China). Fluorescein isothiocyanate (FITC) was obtained from Dingguo Biotechnology Company (Beijing, China).

### Characterization of CPTR and CPTK

The fluorescence spectrum of CPTR and CPTK (50 μM in 10 mM PBS) was measured using a fluorometer (Photon Technology International Inc., Canada) at 0 hour, 24 hours, and 48 hours after being dissolved in PBS. CPTR and CPTK were dissolved in water or DMSO (10 μM). Then, the UV-Vis-NIR spectra were obtained by spectrophotometer (U-4100, Hitachi, Japan).

### Characterization of prodrug-based nanoparticles

To prepare prodrug-based nanoparticles, CPTK and CPTR were dissolved in the deionized water or 10% FBS solution (0.125, 0.25, 0.5, and 1.0 mg/mL), which would self-assemble into nanoparticles based on their amphiphilic structure. The size distribution and zeta potential were measured by Malvern Zetasizer Nano-ZS90 (Malvern Instruments, Malvern, UK) based on dynamic light scattering (DLS) method. The morphology was observed by scanning electron microscopy (SEM, Merlin, Zeiss, Germany).

### Cell lines

HeLa, MCF7, B16F10, 4T1, and CT26 cells were obtained from American Type Culture Collection, Rockville, USA, and cultured in DMEM with 10% (v/v) heat-inactivated FBS, 100 mg/mL streptomycin, and 100 units/mL penicillin in an incubator (37°C, 5% CO_2_, 95% humidity).

### Flow cytometry

CPTR and CPTK were labeled with FITC for cell uptake analysis. CPTR (100 mg) and FITC were dissolved in 30 mL of deionized water with a molar ratio of 1.2:1, and the reaction was conducted at room temperature for 24 hours. Subsequently, insoluble solids in the water were removed by centrifugation (8000 rpm) after sonication. CPTK-FITC was synthesized with similar methods (CPTK: 100 mg). CT26 cells were cultured in 6-well plates at a density of 1.0 × 10^5^ cells per well. After incubation at 37°C for 24 hours, cells were treated with different formulations with equivalent CPT concentration (5 μM), respectively, and then cultured for another 6 hours. The control group was treated with an equal volume of serum-supplemented culture medium without drug. Then, cells were washed with PBS and analyzed through flow cytometry (FACS Caliber, Becton-Dickinson, San Jose, CA, USA).

### Confocal laser scanning microscopy (CLSM)

CT26 cells were seeded on the coverslips in 6-well plates with a density of 1.0 × 10^5^ cells per well and cultured for 24 hours, then CPT, CPTK, and CPTR were added at a concentration of 5 μM. After 6 hours incubation, cells were treated with MitoTracker Red for another 30 minutes at 37°C, then washed and fixed with 3.7% paraformaldehyde, followed by observation using a CLSM, Leica TCS SP2; Leica Microsystems, Wetzlar, Germany.

### Cytotoxicity assay

MTT assay was employed to assess the cytotoxicity of CPT, CPTK, and CPTR against different cell lines, including HeLa, 4T1, B16F10, and CT26 cells. Briefly, cells were seeded at a density of 1.0 × 10^4^ cells per well in 96-well plates and incubated for 24 hours. Then, the culture medium was replaced with a medium containing different concentrations of CPT, CPTK, and CPTR. The CPTK and CPTR were dissolved in saline before use. CPT was first dissolved in DMSO and then diluted in a culture medium. After another 48 hours, 20 μL of MTT (5 mg/mL) was added to each well and the cells were incubated for another 4 hours. Then, MTT-containing solution was removed and 200 μL of DMSO was added to dissolve the MTT formazan crystals. Finally, the absorbance at 490 nm of each well was recorded by an ELISA microplate reader (Bio-Rad, USA). The cell viability (%) was calculated according to the following equation:

Cell viability (%) = (Asample/Acontrol) × 100%

Where, Asample and Acontrol represented the absorbance in the drug-treated groups and control group, respectively. Data were shown as mean ± SD based on triplicate independent experiments.

### Cell apoptosis assay

CT26 cells were cultured in 6-well plates with a density of 1.0 × 10^5^ cells per well and cultured for 24 hours. Then, CPT, CPTK, and CPTR were added with a final concentration of 5 μM. After 24 hours incubation, the apoptotic cells were treated with the Annexin V-FITC apoptosis detection kit according to the manufacturer’s instructions (BD Biosciences, San Jose, USA) and the results were analyzed by flow cytometry.

### Intracellular ROS detection

The ROS generation was accessed by ROS Assay Kit (DCFH-DA) in different cell lines, including 4T1, B16F10, HeLa, and CT26 cells. Briefly, cells were seeded at a density of 1.0 × 10^4^ cells per well in 24-well plates and incubated for 24 hours. Then, the culture medium for each well was replaced with 500 μL of DMEM containing CPT, CPTK, and CPTR at a final drug concentration of 5 μM. After 6 hours incubation, cells were treated with the ROS fluorescent probe (10 mM) for 20 minutes and washed thrice with PBS for CLSM observation.

### Mitochondrial potential assessment

CT26 cells were seeded on 24-well plates at a density of 1.0 × 10^4^ cells per well and treated with 5 μM of CPT, CPTK, and CPTR for 2 hours, respectively. Subsequently, the cells were cocultured with rhodamine 123 for 30 minutes. Then, the cells were washed with PBS and observed with a fluorescence microscope and analyzed by flow cytometry.

### Intracellular Ca^2+^ detection

The Fluo-4 AM probe was applied to access the intracellular Ca^2+^ concentration. Briefly, CT26 cells were seeded on 24-well plates at a density of 1.0 × 10^4^ cells per well and treated with 5 μM of CPT, CPTK, and CPTR for 24 hours, respectively. After being washed with fresh mediums, the cells in different groups were incubated with Fluo-4 AM for 20 minutes and evaluated with flow cytometry.

### 3D spheroid model (3DSM) for *in vitro* tumor penetration

CT26 cells were seeded in low attachment 24-well plates at a density of about 4.0 × 10^4^ cells per well and incubated at 37°C for 5 days. Then, CPT, CPTK, and CPTR were added into the media with the final CPT concentration of 10 μg/mL and incubated for another 2 or 24 hours. Then, the 3DSMs were washed by PBS thrice and put onto a glass slide for CLSM observation.

### Pharmacokinetics

Female BALB/C mice (4-5 weeks) were bought from Vital River Company (Beijing, China). The normal mice were injected with CPT, CPTK, and CPTR at an equivalent CPT dose of 1 mg/kg through tail vein. At different time intervals (0.5, 1, 2, 4, 6, 8, 12, and 24 hours), the blood samples were collected through tail vein using sodium-heparinized micropipettes, transferred to heparinized blood collecting tubes, and stored at −80°C for further analysis. Then, 20 μL of blood was transferred to a black 96-well plate and diluted to 200 μL by PBS. Furthermore, CPT fluorescence was measured on a Tecan infinite M200 Microplate Readers (Tecan, Austria) at λex = 370 nm and λem = 435 nm, and the fluorescence intensity of untreated groups was used as a negative control. The acquired values were normalized to CPT concentration which was calculated based on the naked CPT calibration curves.

### *In vivo* tumor treatment and histological analysis

CT26 cells suspended in PBS were subcutaneously injected into BALB/C mice to establish CT26 tumor-bearing models for investigation of *in vivo* antitumor efficacy. When the tumor size became approximately 150 mm^3^, the tumor-bearing mice were divided randomly into six groups and administered with PBS, CPTK, and CPTR through intravenous or intraperitoneal (for CPT) injection every 3 days, with a total 5 times injection (10 mg/Kg of CPT). The CPTK and CPTR were dissolved in saline before use *in vivo*. CPT was first dissolved in DMSO and then diluted in normal saline. The tumor size and body weight were measured every other day. Tumor volume (V) was calculated as follows: V = 0.5 ab2, where a and b represented the longest (a, mm) and the shortest (b, mm) diameters of tumor measured by a Vernier caliper. After treatment, the heart, liver, lung, spleen, kidney, and tumor were dissected from the mice for histopathological analysis. The tissues were paraffin embedded and sliced at 5 mm thickness. Then, the tissue slices were stained with hematoxylin and eosin (H&E) to assess histological alterations with a microscope.

### Ethical statement

All animal experiments were performed under the Guidelines for Care and Use of Laboratory Animals of Northeast Normal University and approved by the Animal Ethics Committee of Northeast Normal University.

### Statistical analysis

All measurements were conducted in triplicate and expressed as mean ± standard deviation. The Student’s t-test or the one-way ANOVA test was performed to compare the statistical significance. *p < 0.05 was considered statistically significant. **p < 0.01 and ***p < 0.001 were considered extremely statistically significant.

## RESULTS

### Synthesis and characterization of CPTK and CPTR

**FIGURE S1:**
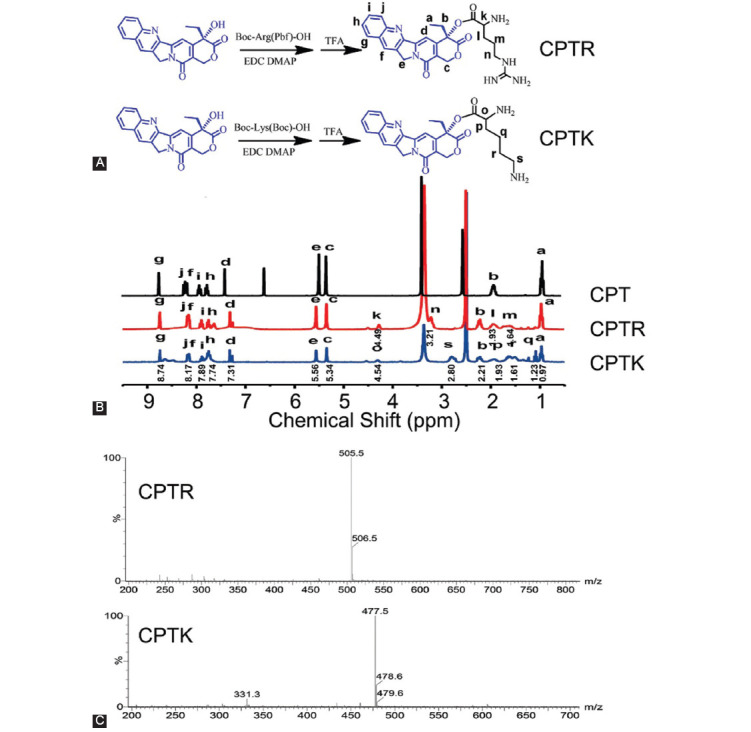
Synthesis and characterization of CPTR and CPTK. (A) Synthesis procedures of CPTR and CPTK; (B) their structures characterized by 1H-NMR spectra; and (C) mass spectrometer. *CPTR was synthesized as our previous work [51].

Water-soluble prodrugs CPTR and CPTK were prepared by the coupling reaction between CPT with Arg or Lys, respectively (Figure S1A). CPTR (percentage yield: ~78.5%) and CPTK (percentage yield: ~87.4%) were characterized by 1H-NMR, and the position of each peak was labeled, as shown in Figure S1. Typically, the peaks from 1.2 ppm to nearly 2 ppm and 4.5 ppm represent that arginine or lysine was successfully conjugated to CPT (Figure S1B). The result of mass spectrometry (Figure S1C) further proved the successful synthesis of CPTR and CPTK.

### Characterization of prodrug-based nanoparticles

**FIGURE 1:**
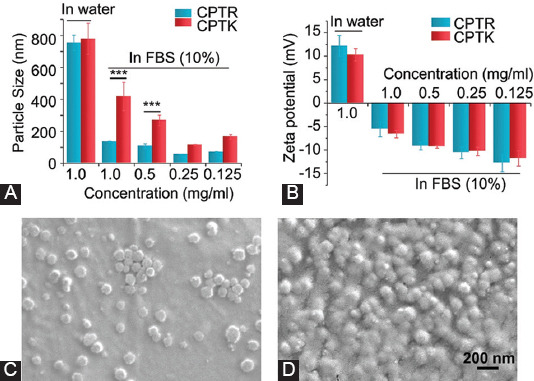
Characterization of CPTK/FBS and CPTR/FBS. (A) Particle size of CPTK and CPTR with different CPT concentration in FBS (10%); (B) zeta potential; and (C and D) the SEM picture of CPTR/FBS and CPTK/FBS. *** *p* < 0.001 (Student's t-test).

The introduction of water-soluble amino acids increased the water solubility of CPT. Meanwhile, their positively charged unit was used for mitochondrial-targeted delivery. However, a high concentration of negatively charged proteins existing in the serum will be absorbed on the surface of the nanosystems based on electrostatic adsorption and affects the *in vivo* fate of nanosystems [28]. Thus, it is necessary to characterize the physicochemical variations of the CPTK/CPTR-based nanosystem after coincubation with negatively charged proteins. For this, CPTK and CPTR were incubated with 10% FBS solution at different drug (CPT) concentrations (0.125-1 mg/mL). As shown in Figure 1, the size of CPTR/FBS was much smaller than that of CPTK/FBS under different drug concentrations. This phenomenon can be attributed to the guanidinium group of arginine, which provides a stronger interaction with aromatic residues in proteins to reduce protein aggregation compared with lysine [29,30]. The zeta potential of CPTR and CPTK in 10% FBS solution was also measured, both showing a sharp decrease after being complexed with FBS (Figure 1B).

**FIGURE S2:**
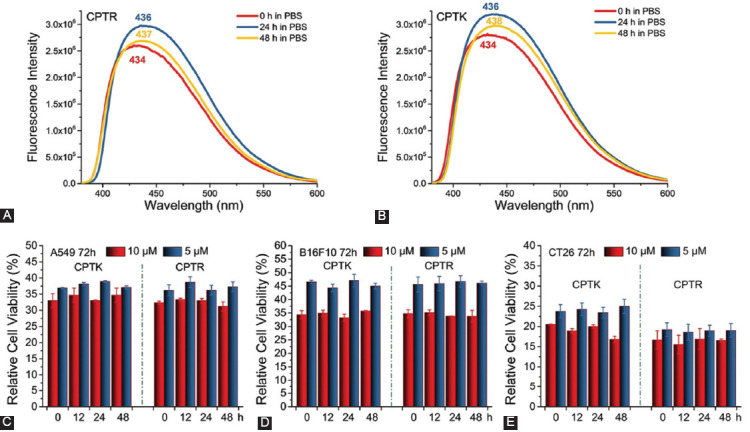
(A and B) Stability of CPTR and CPTK measured by fluorescence spectrum and (C, D and E) MTT analysis in A549, B16F10, and CT26 cells.

The closed lactone form of CPT had highly efficient anti-cancer activity, which decreased significantly in the open lactone form. To estimate the lactone stability of CPTR and CPTK, fluorescence emission spectral shift measurements were performed. As displayed in Figure S2A and B, even after the nanosystems were dissolved in PBS for 48 hours, the emission peak was only shifted from 434 nm to 437 nm for CPTR and from 434 nm to 438 nm for CPTK, which indicated that both CPTR and CPTK were mostly remained in closed lactone form with highly efficient anti-cancer activity, rather than open carboxylate with an emission peak at 446 nm [31,32]. MTT assay in A549, B16F10, and CT26 cell lines also confirmed that there was no obvious difference in the anti-cancer ability of CPTR and CPTK after 0, 12, 24, and 48 hours incubation in PBS, which was further proved the high stability of prodrugs (Figure S2C-E).

**FIGURE S3:**
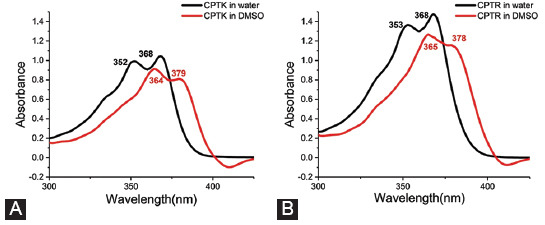
(A and B) UV spectrum of CPTK and CPTR in various solvent (CPT concentration: 10 μM).

To study the self-assembly behavior of the prodrug, the UV-Vis spectrum was analyzed to investigate the state of CPTR and CPTK in water and DMSO. Figure S3A shows that CPTK had two obvious absorption peaks at 352 nm and 368 nm in water, and 367 nm and 370 nm in DMSO, respectively, indicating the existence of an aggregated state. Similar properties can also be observed in CPTR (Figure S3B), and the self-assembling process might be attributed to the hydrophobic and π-π stacking interactions based on the π-electronic planar of CPT [33,34].

### *In vitro* cytotoxicity and cell uptake efficiency

**FIGURE 2:**
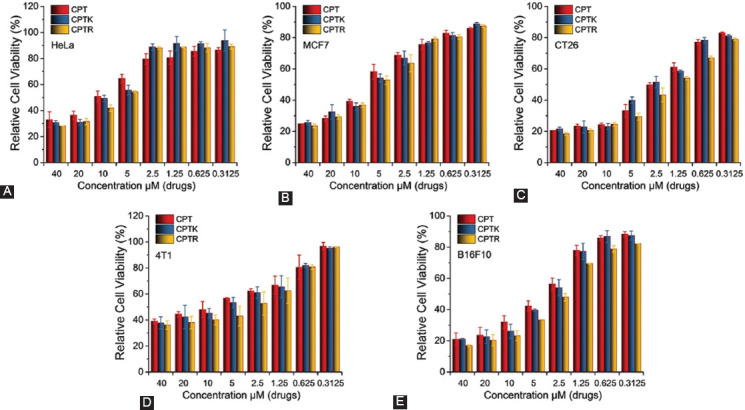
*In vitro* cytotoxicity of CPT, CPTK, and CPTR in different cell lines. (A) HeLa cells; (B) MCF7 cells; (C) CT26 cells; (D) 4T1 cells; and (E) B16F10 cells. Cell viability was evaluated by MTT assay after different treatments for 48 hours.

**FIGURE 3:**
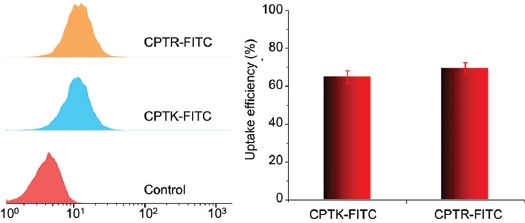
Cellular uptake in CT26 cell, CPTK, and CPTR labeled with FITC (6 hours, 5 μM).

The fluorescence spectrum had shown that CPTK and CPTR were able to keep closed lactone for a long time, which was considered an active form of CPT. The anti-cancer ability of CPT, CPTK, and CPTR was measured in HeLa, MCF7, CT26, 4T1, and B16F10 cells (Figure 2). CPTR showed a higher cell proliferation inhibition rate compared with CPT and CPTK. No significant differences in cell viability were found among the three groups. However, the IC50s of CPTR in different cell lines were lower than in the other two formulations, as shown in Table S1. To explore the underlying mechanism, we selected CT26 cells to study the cellular uptake efficiency of CPTK and CPTR. CPTK and CPTR were labeled with FITC. The result showed that CPTK and CPTR had similar cellular uptake, which means that cellular uptake was not the key factor that led to the difference in cell toxicity (Figure 3).

### Cell apoptosis, ROS detection, mitochondria CPT colocalization assay, and associated membrane potential assessment

**FIGURE 4:**
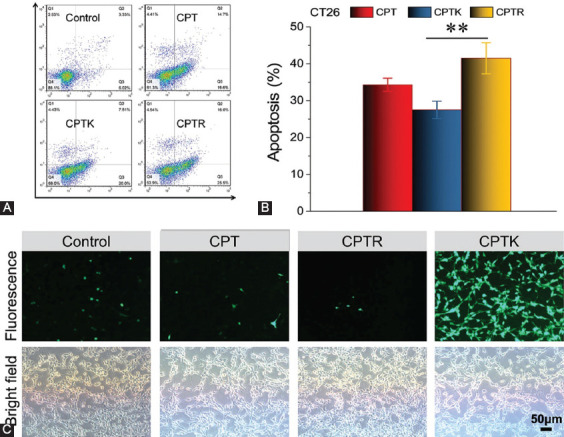
Measurement of apoptosis rates of CT26 cells treated with CPT, CPTK, or CPTR and the ROS detection. (A) Flow cytometry analysis of apoptosis (24 hours, 5 μM); (B) apoptosis rate (24 hours, 5 μM); and (C) ROS generation after CPT, CPTR, and CPTK treatment (6 hours, 5 μM). The statistical significance was calculated through one-way ANOVA test. **p < 0.01.

**FIGURE S4:**
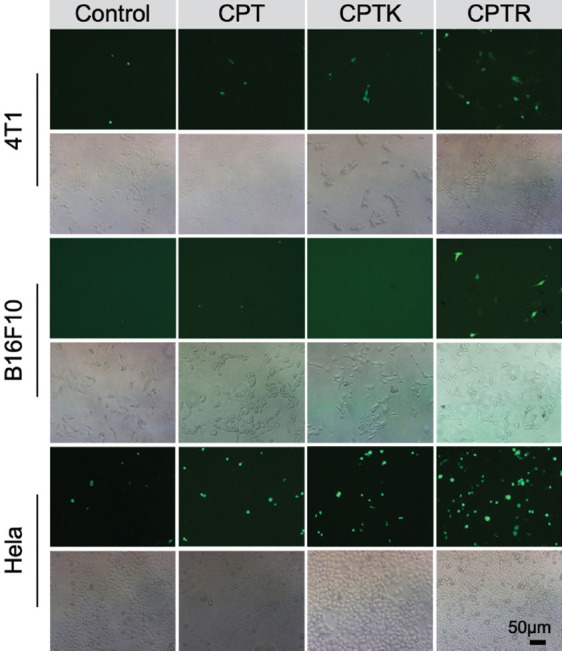
ROS detection in 4T1, B16F10, and HeLa cells (Bright field, green fluorescence) (5 μM).

Cell apoptosis assay was further employed to evaluate the anti-cancer activity of CPT, CPTK, and CPTR. At an equivalent drug concentration of 5 μM, an increase in cell apoptosis (41.5%) was found in the CPTR group compared with CPT (34.3%) and CPTK (27.5%) (Figure 4A and B, control group: ~9.35%), which is different from the above results of cellular uptake. Recent studies proved that CPT inhibited mitochondria oxygen consumption and increased ROS generation, which contributed to cell apoptosis [13,14]. The ROS detection was conducted in CT26 cells, as shown in Figure 4C, and the CPTR group exhibited a higher ROS level (green fluorescence signal). Similar results were obtained in other cell lines (Figure S4).

**FIGURE 5:**
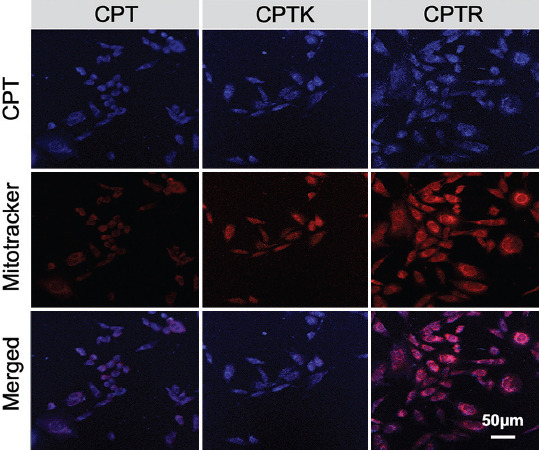
CLSM images of colocalization of mitochondria and CPT (6 hours, 5 μM). Blue color represents CPT, CPTK, or CPTR. Mitochondria were stained with MitoTracker Red (red).

Then, the intracellular fate of CPT, CPTK, and CPTR was explored. As shown in Figure 5, CLSM images revealed that CPTR showed significant mitochondrial colocalization signal despite similar cellular uptake, which confirmed that the efficient anti-cancer ability of CPTR was associated with mitochondria-targeted delivery and subsequent ROS generation, except for the function of topoisomerase I inhibition for CPT.

**FIGURE 6:**
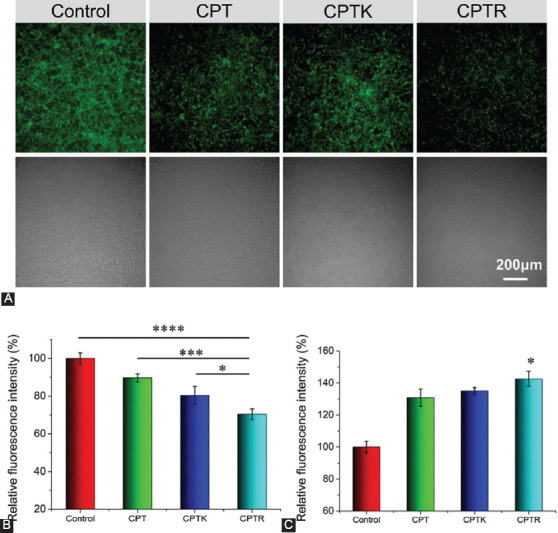
Variations in mitochondrial and calcium homeostasis. (A) CLSM images of mitochondrial potential changes (2 hours, 5 μM); (B) flow cytometry analysis of mitochondrial membrane potential changes (2 hours, 5 μM); and (C) evaluation of calcium ion concentration (24 hours, 5 μM). *p < 0.05, ***p < 0.001, ****p < 0.0001 (one-way ANOVA).

The membrane potential variation was also accessed after 2 hours treatment of CPT, CPTK, and CPTR. As shown in Figures 6A and B, the mitochondrial membrane potential in CPTR decreased significantly when compared with the untreated group. These results verified the prediction that mitochondria-targeted delivery induces significantly mitochondrial dysfunction. Furthermore, mitochondria are involved in intracellular calcium homeostasis regulation, which is attractive for therapeutic intervention [35]. The changes in cellular Ca^2+^ concentration were accessed due to its important role in tumors [36]. As shown in Figure 6C, Ca^2+^ in the CPTR group was increased compared with the control group (*p* < 0.05). Although the calcium concentration in the CPTR group was higher than that in the CPT and CPTK groups, the data were not statistically different. This may be related to the toxicity of CPT itself.

### *In vitro* tumor penetration to 3DSM

**FIGURE 7:**
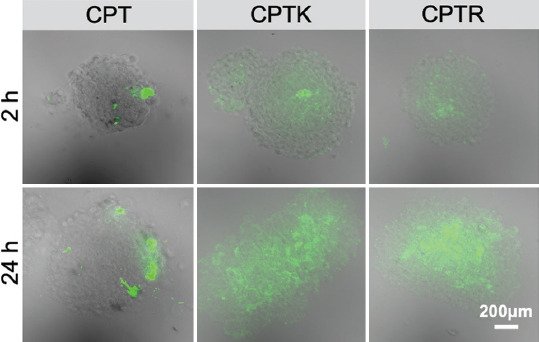
*In vitro* tumor penetration of CPT, CPTK, and CPTR in 3DSMs after 2 or 24 hours of incubation (with the CPT concentration of 10 μg/mL). Scale bar represents 200 μm.

The *in vitro* evaluation of tumor penetration ability of CPT, CPTK, and CPTR was carried out using a 3DSM derived from CT26 cells. As shown in Figure 7, CPT was mostly dispersed at the periphery of 3DSM, and the negligible fluorescence signal was detected in the center of 3DSM after 2 hours incubation. By contrast, the fluorescence signals of CPTK and CPTR were detected much deeper inside of 3DSM. After 24 hours coincubation, the stronger fluorescence signals for CPTK and CPTR suggested their higher tumor penetration ability than CPT.

### Pharmacokinetics, *in vivo* tumor treatment, and histological analyses

**FIGURE 8:**
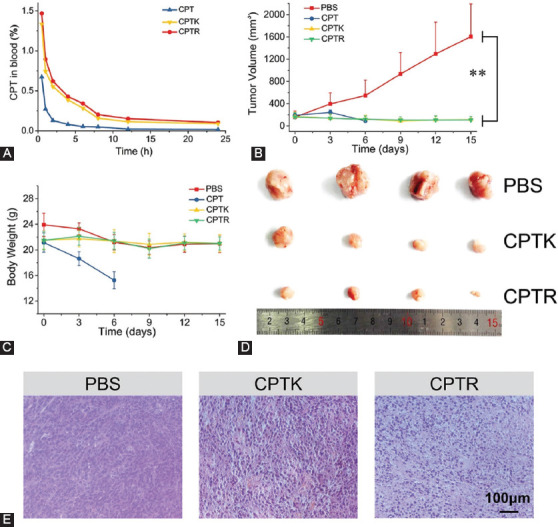
*In vivo* anti-tumor therapy. (A) CPT concentration in blood versus time after intravenous injection of CPT, CPTK, and CPTR in mice (10 mg/Kg of CPT concentration); (B) tumor volume changes during treatment (0, 3, 6, 9, and 12 were the time point of drug injection); (C) the body weight changes during treatment; (D) tumors were excised from each group after the sacrifice of mice; and (E) H&E staining images of tumor collected from CPTK and CPTR injected mice and control treated mice with PBS. ** *p* < 0.01 (one-way ANOVA).

The blood concentration of CPT at fixed time intervals was further measured to study the pharmacokinetic profiles of different formulations (Figure 8A). Consistent with previous reports, the detected CPT concentration in blood decreased dramatically. However, the decrease in CPT concentration was significantly delayed in CPTK and CPTR groups. The improved blood concentration of CPTK and CPTR may be attributed to the positive charge, which was combined with negatively charged serum proteins after injecting into the blood, thus preventing the formation of large particle embolism inducing mice death.

Inspired by the *in vitro* experiments, *in vivo* anti-cancer evaluation was carried out to further investigate the potential use of CPTK and CPTR. As displayed in Figure 8B and D, the growth rate of tumors was significantly restrained in all treatment groups, and the average tumor size was no larger than 500 mm^3^. The groups of CPTK and CPTR showed high antitumor efficacy with an average tumor size lower than 150 mm^3^ after treatment, which was in accordance with the cell apoptosis results. In addition, mice treated with CPT had a sharp decrease in body weight compared to other groups, indicating that CPT had serious side effects on mice when injected directly (Figure 8C).

**FIGURE S5:**
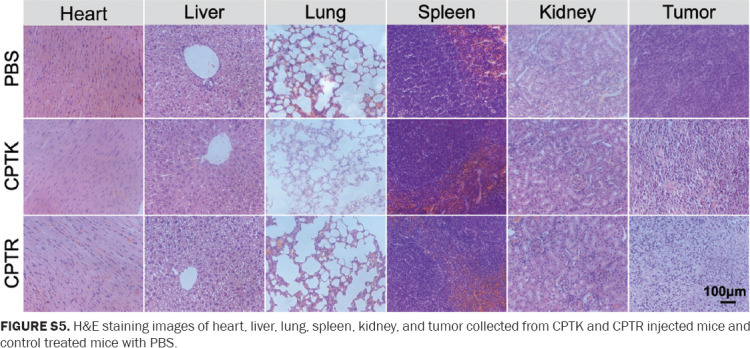
H&E staining images of heart, liver, lung, spleen, kidney, and tumor collected from CPTK and CPTR injected mice and control treated mice with PBS.

Further safety evaluation of CPTK and CPTR was analyzed through H&E staining (Figure 8E and Figure S5). Compared with the PBS group, the CPTK and CPTR groups showed obvious cell nuclei dissolution, especially in CPTR groups. H&E-stained tissue sections from the heart, liver, spleen, lung, and kidney demonstrated that the prodrug had no significant damage or toxicity to these organs. According to *in vivo* antitumor therapy results, CPTR and CPTK showed high efficiency in tumor suppression, which may attribute to their deeper tumor penetration. More importantly, the positively charged units of CPTR and CPTK had little effect on their blood circulation, which is beneficial for further application.

## DISCUSSION

The clinical application of CPT is limited by poor water solubility, undesirable adverse effects, as well as the instability of lactone rings in the physiological environment. Self-assembly nanocarriers based on prodrugs provided a powerful way for elevating therapy efficiency of CPT-based cancer treatment due to many advantages, including high loading capacity, precisely defined chemical structure, and convenient preparation. For example, CPT can be conjugated with polymers [37], amino acids [38], peptides [39], and polysaccharides [40] to form carrier-free nanomedicine. Among them, the introduction of amino acids to CPT has been regarded as a promising method for modification and further application of CPT due to commercial availability, fewer safety issues, and targeting influx transporters, which have been widely applied to improve pharmaceutical properties of marketed drugs [41]. Herein, positively charged amino acids (lysine and arginine) were introduced to synthesized CPTK and CPTR for the construction of CPT-based drug delivery systems with high CPT loading content and increased stability of lactone ring. Studies have shown that the cationic prodrugs CPTK and CPTR can assemble into uniform nanoparticles with the negatively charged proteins in the blood. Meanwhile, both CPTK and CPTR have excellent lactone stability even after 48 hours, which enjoy highly efficient anti-cancer activity when compared with opened carboxylate form.

In addition to the increased water solubility and stability, due to the positive charge of lysine and arginine, the nanosystems achieved the mitochondrial-targeted delivery of CPT. Since CPT can inhibit mitochondrial oxygen consumption, which leads to elevated ROS levels and final cell apoptosis, the targeted delivery of CPT would contribute to enhanced cancer cells inhibition. Although CPTK and CPTR exhibited similar cellular uptake, CPTR induced more cell apoptosis, which was associated with its better mitochondrial targeting ability. It could be inferred that the topoisomerase I inhibition, the elevated ROS level, and calcium ion concentration contribute to more effective cancer cells inhibition. Upregulation of intracellular calcium concentration to disrupt mitochondrial calcium homeostasis has been recognized as a potential cancer therapy [42].

However, as an important regulator of intracellular homeostasis, mitochondrial changes can also adversely affect intracellular calcium ion concentration. CPTR effectively increases cellular ROS levels by targeting mitochondria, which would stimulate the release of calcium ions from the endoplasmic reticulum [43-45]. Furthermore, the modification of amino acids provides higher tumor penetration ability, which makes CPTK and CPTR show higher cancer suppression ability than CPT. Meanwhile, the side effects of CPTK and CPTR were significantly reduced when compared with free CPT. Free CPT has significant toxic effects due to systemic delivery and the limited therapy efficiency associated with the unstable lactone ring [46].

CPTK and CPTR self-assemble into nanoparticles in the physiological environment, which can passively target tumors. *In vitro* experiments showed that CPTK and CPTR had higher lactone ring stability and fully penetrated tumors when compared with free CPT, which enabled them to effectively inhibit tumor progression with reduced systemic toxicity. Therefore, the introduction of amino acid-modified CPT prodrugs can make up for the shortcomings of current CPT drugs and promote their clinical applications. In cell experiments, it is interesting that an elevated MitoTracker fluorescence level was observed in the CPTR group, which could be associated with mitochondrial biogenesis. Possible reasons involve complex regulation to maintain intracellular homeostasis. On the one hand, oxidative stress-induced mitochondrial damage may activate cellular self-regulation to protect mitochondrial integrity and quality [47]. In this process, cells promote autophagy of damaged mitochondria and stimulate healthy mitochondria to proliferate through mitochondrial biogenesis [48,49]. On the other hand, ROS production induces endoplasmic reticulum stress and subsequent calcium release, which disrupts calcium homeostasis and activates mitochondrial biogenesis [43,44,50]. Since mitochondrial biogenesis plays an important role in tumorigenesis and escape, mitochondrial biogenesis should be considered to optimize therapeutic efficacy in future studies.

## CONCLUSION

In this work, CPTK and CPTR were developed and successfully synthesized to improve both the water solubility and stability of CPT. CPTK and CPTR were also proved to be in closed lactone form, even when dissolved in PBS for 48 hours, which was crucial for keeping their anti-cancer activity. Importantly, CPTR exhibited a significantly higher ability to induce cell apoptosis than CPT and CPTK based on its mitochondria-targeted delivery to increase ROS generation. The *in vivo* anti-cancer results showed that CPTR had a higher tumor-suppressive effect than other groups. Meanwhile, none of them caused toxicity in major organs. Therefore, CPTR can be regarded as a promising candidate prodrug of CPT for clinical antitumor treatment.

## Figures and Tables

**TABLE S1:**
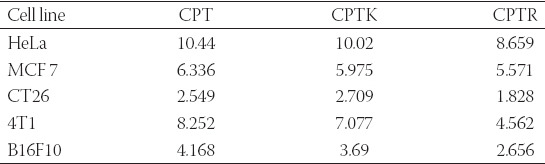
IC50 (μM) of CPT, CPTK, and CPTR in different cell lines.

## SUPPLEMENTAL DATA

METHODS

Synthesis of CPTR and CPTK

CPTR and CPTK were synthesized using a similar method. Briefly, for the preparation of CPTR, 300 mg of CPT, 679 mg of Boc Arg(Pbf )-OH, 496 mg of EDC·HCl, and 315 mg of DMAP were added into the reaction bulb, then 25 mL dichloromethane (DCM) was added and the reaction was conducted at room temperature for 24 hours until the reaction solution became clean. The solution was condensed and transferred to silica column chromatography, and DCM: methanol (95:5, v/v) was employed as the eluent to obtain purified pale yellow powder Boc Arg(Pbf )-CPT. After that, Boc Arg(Pbf )-CPT was dissolved into TFA for about 3 hours to get CPTR. For CPTK, Boc-Arg(Pbf)-OH was replaced with 597 of mg Boc-Lys(Boc)-OH. The other steps are the same as those of CPTR. The structure and molecular weight of CPTR were determined by 1H-NMR spectra, recorded on an AV-300 NMR spectrometer (Bruker, Karlsruhe, Germany) and mass spectrometer (Micromass, Waters Corp., Milford, MA, USA).
